# Gestational Exposure to Sodium Valproate Disrupts Fasciculation of the Mesotelencephalic Dopaminergic Tract, With a Selective Reduction of Dopaminergic Output From the Ventral Tegmental Area

**DOI:** 10.3389/fnana.2020.00029

**Published:** 2020-06-05

**Authors:** Ágota Ádám, Róbert Kemecsei, Verónica Company, Raquel Murcia-Ramón, Iris Juarez, László I. Gerecsei, Gergely Zachar, Diego Echevarría, Eduardo Puelles, Salvador Martínez, András Csillag

**Affiliations:** ^1^Department of Anatomy, Histology, and Embryology, Faculty of Medicine, Semmelweis University, Budapest, Hungary; ^2^Institute of Neuroscience (UMH-CSIC), University of Miguel Hernández, Alicante, Spain

**Keywords:** nigrostriatal pathway, ventral tegmentum, axonal growth, fasciculation, ASD

## Abstract

Gestational exposure to valproic acid (VPA) is known to cause behavioral deficits of sociability, matching similar alterations in human autism spectrum disorder (ASD). Available data are scarce on the neuromorphological changes in VPA-exposed animals. Here, we focused on alterations of the dopaminergic system, which is implicated in motivation and reward, with relevance to social cohesion. Whole brains from 7-day-old mice born to mothers given a single injection of VPA (400 mg/kg b.wt.) on E13.5 were immunostained against tyrosine hydroxylase (TH). They were scanned using the iDISCO method with a laser light-sheet microscope, and the reconstructed images were analyzed in 3D for quantitative morphometry. A marked reduction of mesotelencephalic (MT) axonal fascicles together with a widening of the MT tract were observed in VPA treated mice, while other major brain tracts appeared anatomically intact. We also found a reduction in the abundance of dopaminergic ventral tegmental (VTA) neurons, accompanied by diminished tissue level of DA in ventrobasal telencephalic regions (including the nucleus accumbens (NAc), olfactory tubercle, BST, substantia innominata). Such a reduction of DA was not observed in the non-limbic caudate-putamen. Conversely, the abundance of TH+ cells in the substantia nigra (SN) was increased, presumably due to a compensatory mechanism or to an altered distribution of TH+ neurons occupying the SN and the VTA. The findings suggest that defasciculation of the MT tract and neuronal loss in VTA, followed by diminished dopaminergic input to the ventrobasal telencephalon at a critical time point of embryonic development (E13-E14) may hinder the patterning of certain brain centers underlying decision making and sociability.

## Introduction

Owing to the high and growing incidence of autism spectrum disorder (ASD), this highly complex neurodevelopmental syndrome has been a focus of the investigation. In addition to the numerous clinical and neuropathological studies that analyze ASD, systematic attempts have been made to find suitable animal models for experimental analysis of the molecular mechanisms potentially leading to ASD. One frequently used model in rodents that evolved in the recent decade is the administration of 2-propylpentanoic acid, a.k.a. valproic acid (VPA), a commonly used anticonvulsant, antiepileptic and mood stabilizer, to pregnant females, bringing about characteristic deficits of social behavior in the offspring, *postpartum* (Rodier et al., 1996; Schneider and Przewlocki, 2005; Wagner et al., 2006; for recent reviews see Roullet et al., 2013; Nicolini and Fahnestock, 2018). Impairment of social adaptation and behavior are characteristic features of all forms of ASD. Accordingly, *in utero* exposure to VPA causes sociability deficits which become manifest postnatally, often in the adult animals (Kim et al., 2011; Moldrich et al., 2013; Roullet et al., 2013). VPA-treated animals also exhibit anxiety, depression-like behavior, and abnormal nociception thresholds (Wu et al., 2017). The validity of embryonic exposure to VPA as a model is not restricted to mammals, as evidenced by recent studies in fish (Baronio et al., 2017; Chen et al., 2018) and birds (Nishigori et al., 2013; Sgadò et al., 2018; Lorenzi et al., 2019; Zachar et al., 2019).

The model is in line with the notion that only a minority of human autism cases are attributable to known genetic components (Scherer and Dawson, 2011). Thus, the bulk of ASD cases are supposed to be brought about by environmental or epigenetic factors exerting their effect at critical time points of embryonic development. VPA has long been suspected to have teratogenic effects, including an elevated risk of ASD (Christianson et al., 1994; Chomiak et al., 2013; Christensen et al., 2013), which is why its administration to pregnant mothers is not recommended (Tureci et al., 2011). Teratogenicity of the agent has been described extensively, both in chicken embryos (Whitsel et al., 2002) and in comprehensive human clinical studies (Jentink et al., 2010). These effects have been ascribed mostly to the fact that VPA is an inhibitor of histone deacetylase, influencing transcription (Göttlicher et al., 2001; Kataoka et al., 2013; Moldrich et al., 2013). Just how this propensity of the molecule can be linked to specific defects in autism is yet to be understood but, in any case, the time window of action is a critical issue.

When compared to the multitude of behavioral studies concerned with the maternal challenge to VPA, the available data on the neuromorphological correlates of VPA treatment are remarkably scarce. One possible reason for the apparent reluctance could be that the VPA-elicited behavioral alterations are rather subtle, and all major brain functions look relatively normal. Thus, it might be unlikely that they are accompanied by frank structural defects. Our point of departure was that whatever changes might take place as a result of *in utero* VPA administration, these must be associated with critically timed events of CNS development and they must affect those forebrain systems participating in, or linked to, the social brain network (Goodson, 2005; O’Connell and Hofmann, 2011). In previous studies, E12–15 proved to be the ideal time frame for VPA administration in rats or mice (Kataoka et al., 2013), as injections before this time often cause massive and non-specific teratogenic alterations, while treatments later than this are ineffective or inconclusive. Remarkably, this time window coincides with the appearance of dopaminergic cells in the brainstem, as revealed by labeling of tyrosine hydroxylase (TH), the rate-limiting enzyme of DA synthesis, and the early development of the dopaminergic axons that later form the mesotelencephalic (tegmentostriatal) pathway. It seems reasonable to assume that VPA might interfere with this process and, by so doing, with the development and pattern formation in the target regions of the mesotelencephalic pathway. Prenatal exposure of mice to VPA was shown to cause a reduction of dendritic spine density in the layers II/III and V of the prefrontal cortex, and also in the CA1 area of the hippocampus. These alterations could be antagonized by chronic treatment with AHDH drugs, promoting dopamine release (Hara et al., 2016).

Of these targets, the ventrobasal forebrain, including the nucleus accumbens (NAc), receives dopaminergic input mainly from the ventral tegmental area (VTA), whereas the dorsolateral striatum or caudate-putamen (CPu) receives input mainly from the substantia nigra (SN), pars compacta (SNc). Due to its composition and to the extensive connections with limbic forebrain centers (ventral pallidum, extended amygdala, septum, bed nucleus of stria terminalis, olfactory tubercle, prefrontal cortex, and certain hypothalamic nuclei) the ventrobasal forebrain occupies a central position in decision-making based on learned associations, reward, social attraction or repulsion, as well as reproductive cues (courtship, mating, care for offspring). Most of these functions are also relevant to and maybe perturbed in, ASD. Despite an impressive number of studies focusing on the pre- and postnatal development of the dopaminergic neurons and their pathways (see below), the developmental dynamics of the ventrotegmental–accumbens (mesolimbic) pathway is less well understood than its partner system (nigrostriatal).

Dopamine is known to influence the development and maturation of target neurons, as evidenced by co-culture studies (Snyder-Keller et al., 2008). Intact, well-balanced functioning of the ventrobasal forebrain, constituting part of the social brain network, likely requires a complex developmental process in which the relationships of dopaminergic neurons to their targets (dopaminoceptive neurons) are assembled and matured. Should this process fail (or be retarded) due to a deficiency of DA input at a critical time point of embryonic development, the individual may end up showing weaker or inadequate response to social signals *postpartum*.

Based on the above working hypothesis, we posed the following questions:

(i) Are there any differences between the quantitative morphometric parameters of the midbrain dopaminergic centers and pathways of mouse pups born to VPA-exposed mothers and of those born to vehicle-injected mothers?
(ii) Are there any differences between the concentration of DA in ventrobasal forebrain (NAc) and striatal (CPu) tissue extracted from mouse pups born to VPA-exposed mothers and of those born to vehicle-injected mothers?

To answer the first question, we decided to employ a recently developed method of whole-mount fluorescence immunocytochemistry against TH, combined with tissue clearing. The foundations of these techniques were laid in the last decade (Dodt et al., 2007; Ertürk et al., 2012), and then further optimized for brain pathway analysis (Belle et al., 2014). The result was a rapid procedure of immunostaining and tissue clearing, combined with light-sheet microscopy for volumetric imaging (iDISCO method, Renier et al., 2014, for a recent overview see Godefroy et al., 2019). As applied in the present study, the iDISCO method enables visualization and spatial rotation of the regions of interest in 3D, without truncation or distortion, all being critical factors for an unbiased stereological approach. On the other hand, we had to accept certain technical limitations, as evident from literature and our preliminary experiments: for an ideal penetration of antibodies the brain size had to be kept small (while still sufficiently mature for visualization of the regions of interest). Thus, we opted for processing and analyzing the brains of 7-day-old (P7) mice. The added bonus of using this age is low myelin content, which enabled optimal tissue clearing.

Concerning the second question, we utilized the ELISA method for the rapid and simultaneous assay of DA in tissue samples of NAc and CPu dissected from P7 mice. This approach allowed the neurochemical analysis of brain tissue to be performed as an independent study in mice of that were the same age as those used for morphometry.

The possibility that ASD might be associated with defective development of the dopaminergic system, has already been raised as a logical suggestion (Bissonette and Roesch, 2016; Alsanie et al., 2017). The present study represents a definite step in this direction, by demonstrating distinct and coherent neuroanatomical and neurochemical changes in the dopaminergic system of mice which were evoked by maternal VPA challenge. Besides, the results may also have some translational value toward other major neuropsychiatric syndromes associated with selective impairment of dopaminergic circuits [such as Parkinson’s disease (PD)].

## Materials and Methods

### Experimental Animals

We used standard laboratory mice of the C57BL/6J strain for most of the experiments. For the experiments involving maternal exposure with VPA, the animals were obtained from the laboratory animal breeding facility of the Institute of Oncology, Budapest. Further processing of this cohort of animals took place in the animal facility of the Department of Anatomy, Budapest. Following mating, the females were checked for vaginal plugs and the first midday with a detectable plug was designated as E0.5, and the day of birth as P0. Mothers were treated with VPA or saline (controls). For the first series of measurements on whole-mount brains, four litters with a total of 29 pups of mixed sex were used, 11 controls (CTR), and 18 VPA exposed (VPA) animals. Of these, 17 were perfused on postnatal day 7 (P7) and 12 were perfused on P21. Thereafter, having processed three brains of the older group, these specimens proved suboptimal for our purpose, due to an incomplete clearing in the course of the iDISCO procedure. Therefore, we decided to focus on P7 animals throughout the study. Twelve animals (six VPA and six CTR) were used for systematic comparison of TH+ structures in whole-mount specimens, whereas the rest of the perfused P7 brains were used for preliminary experiments, optimizing the iDISCO procedure, testing of the antibodies and for routine immunohistological staining on serial sections (both coronal and sagittal).

Another cohort of mouse pups (seven males and five females) from three litters obtained from the animal facility of the Institute of Neuroscience, Alicante was used for estimation of sexual dimorphism in the quantitative parameters of TH+ structures, in CTR animals at P7, by applying the same iDISCO protocol.

The transcription factor TTF-1 (Nkx2.1) is known to participate in the guidance of the axons in the mesotelencephalic tract (to be discussed below). For analysis of the relationship between the distribution of TH+ structures and this transcription factor, we used a reporter and a Cre recombinant mouse strain (C57BL/6 background), resulting from a tdTomato reporter line (Rosa^tdTomato^ flox mice; Madisen et al., 2010) crossed with hemizygous mice expressing Cre recombinase driven by Nkx2.1 promoter (Nkx2.1^Cre^ mice; The Jackson Laboratory, #008661), in the animal facility of the Institute of Neuroscience, Alicante. Breeding was performed as follows: Nkx2.1^Cre/+^ males were crossed with Rosa^tdTomato/tdTomato^ females. Pregnant transgenic females were injected with VPA or saline, as described above. Notably, these animals were particularly sensitive to VPA and displayed low birth rates. Therefore, these embryos were removed at E18.5 by caesarian section, yielding a total of two VPA exposed and 10 CTR specimens, which were used for qualitative comparison, using fluorescent microscopy on vibratome sections.

The cohort of C57BL/6 mice used for the DA assays was bred and treated at the animal facility of the Anatomy Department, Budapest. A total of 22 pups (11 males and 11 females) from five litters were used. The pregnant mothers were injected with VPA or saline, as described above. The brain samples for the ELISA assay were dissected in four sessions from sexed P7 pups.

The animals were kept at 24°C under a 12:12 h light:dark cycle with food and water available *ad libitum*. The animal housing conditions corresponded to international standards. The experiments were conducted in conformity with the laws and regulations controlling experiments and procedures in live animals, as described in the Principles of Laboratory Animal Care (NIH Publication 85–23, revised 1985). The Department of Anatomy of Semmelweis University, Budapest has a valid experimental license for working with laboratory animals including mice, issued by the Food Chain Safety and Animal Health Directorate of the Government Office for Pest County, Hungary (license number: XIV-I-001-2269-4/2012). At the Neuroscience Institute in Alicante, all mouse manipulation and experimental procedures were performed according to the directives of the Spanish and European Union governments, and the protocols were approved by the Universidad Miguel Hernández OEP Animal Experimentation Committee (code 2014/VSC/PEA/00166).

### Treatment With VPA

For whole-mount histology of young postnatal animals, pregnant females were housed under standard laboratory conditions. On the day 13.5 of gestation, the animals were weighed and given a single hypodermic injection of 400 mg/g b.wt sodium valproate, freshly dissolved in physiological saline (VPA animals), or an equivalent volume of the vehicle for controls (CTR animals), into the occipital skin (we preferred not to give intraperitoneal injections to pregnant mothers). Once born, the pups were kept together with the mothers until they had reached day 7 of age. Then they were perfused under terminal anesthesia (intraperitoneal injection of 460 mg/kg b.w. ketamine and 80 mg/kg b.w. xylazine) with 4% paraformaldehyde through the left ventricle of the heart, the brains were removed and postfixed in paraformaldehyde until further processing.

In the case of transgenic animals, pregnant females were treated with VPA or vehicle as above on E13.5, and the embryos were removed by caesarian section at E18.5. Briefly, the pregnant dams were killed by cervical dislocation, the uterine horns were removed and immediately placed in ice-cold buffer (PBS) solution. The embryos were individually dissected from the amniotic sac, the heads cut off and left in 4% paraformaldehyde (PBS) under gentle agitation at 4°C for 48 h. Then the brains were dissected under a binocular stereomicroscope and kept in PBS (with 0.1% Na-azide) at 4°C. Only those brains with a detectable fluorescence from tdTomato (viewed under a Leica stereomicroscope) were processed further.

### Whole-Mount Immunocytochemistry and Tissue Clearing (iDISCO)

Essentially, we followed the procedure by Renier et al. (2014) with modifications. The postfixed whole brains were first washed in PBS for 2 days, under agitation, followed by dehydration in 25%, 50%, 75%, and 100% methanol in PBS (90 min each). They were then bleached with 3% H_2_O_2_ in 100% methanol overnight. Brains were rehydrated in 100%, 75%, 50%, and 25% methanol (in PBS), allowing 90 min for each step. For blocking and permeabilization, the pretreated brains were incubated in PBS-GT (PBS, 0.2% gelatin, and 0.5% Triton-X-100) containing sodium azide (0.1%) for 3 days at room temperature under agitation. Then they were treated with the primary, anti-TH antibody (rabbit polyclonal IgG, Merck Millipore, catalog# AB152) at 1:500 concentration in PBS-GT for 7–10 days at 37°C in a humidified incubator. Brains were washed in PBS-GT for 1 day (changing solutions 7–8 times) and incubated for 24 h at 37°C with the secondary antibody (goat anti-rabbit Cy5; emission color: far-red—649–670 nm, dilution 1:500, Invitrogen, catalog# A10523) in PBS-GT. The solution containing the secondary antibody was previously filtered through a 0.2 μm cellulose acetate sterile syringe filter. After the incubation with the secondary antibody, and before the clearing, the brains were washed again in PBS-GT for 1 day (changing solutions 7–8 times). This was followed by dehydration in methanol (50% in PBS, 80% in distilled water, and 100% 2×, 1.5 h for each change) and clearing in a 1:2 mixture of methanol/dichloromethane (DCM; Sigma-Aldrich, catalog# 270997) overnight. In all the steps following the secondary Ab, the specimens were protected from light by wrapping the vials in aluminum foil. For the final clearing procedure, the brains were incubated in 100% DCM for approx. Two hours at room temperature, until they sank to the bottom of the vial. Finally, the brains were transferred to 100% dibenzyl ether (DBE; Sigma-Aldrich, catalog# 108014) for at least 30 min (changed twice) before viewing and imaging.

The cleared P7 brains were examined with an Ultramicroscope (LaVision BioTec, Bielefeld, Germany) equipped with an Olympus MVX10 objective lens system. The brains were mounted on a specimen holder to maintain a standard and reproducible position while being immersed into the imaging chamber filled with ethyl cinnamate 99% (Sigma-Aldrich, catalog # 112372). The brain was positioned on its ventral surface, with the rostrocaudal axis orthogonal to the path of the scanning beam. Thus, the tomographic scans from which the compound images were generated corresponded to horizontal brain sections. Images were acquired by ImSpectorPro software (LaVision BioTec). The step size in Z-orientation between each image was fixed at 7 μm for 1.25× magnification.

### Image Processing, Cell Counting

3D images of the CNS were reconstructed from a Z-series of ultramicroscope fluorescence images using the image analysis software package IMARIS, version 8.3.0_64^1^. For quantitative morphometric analysis, the regions of interest (ROI) were selected by visual observation and enclosed within a rectangular cuboid (reference space) superimposed over the image on the screen.

**Figure 1:**
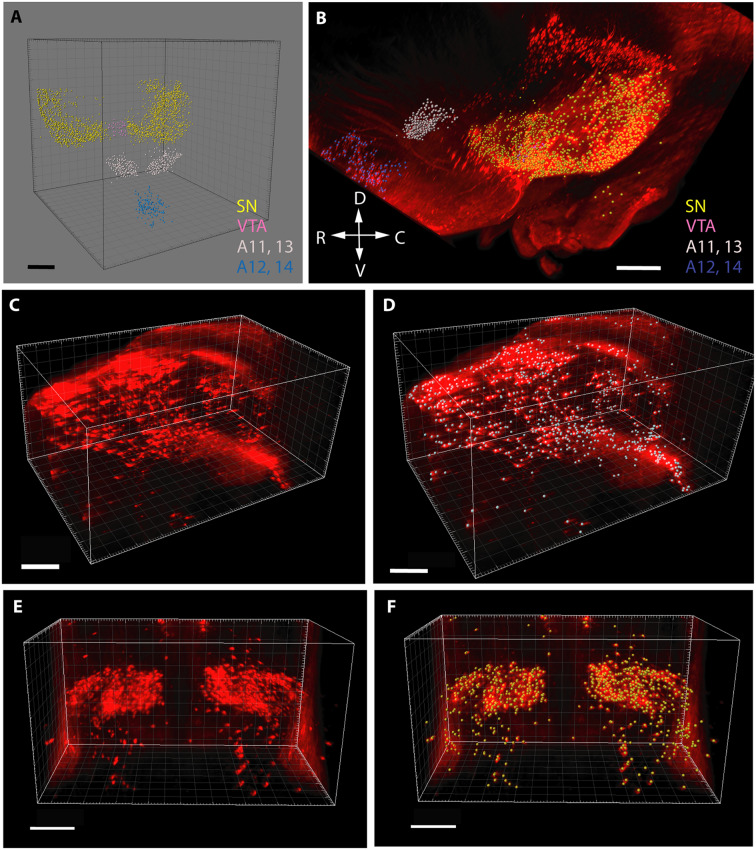
Segmentation and volume rendering of catecholaminergic diencephalic and mesencephalic cell groups with IMARIS, version 8.3.0, on images from representative control brain specimens immunostained for tyrosine hydroxylase (TH), using the iDISCO method. **(A)** Schematic representation of the false colors used for the demonstration of catecholaminergic structures, after digital subtraction of tissue background. **(B)** Color-coded catecholaminergic structures shown in their original three-dimensional position (SN—substantia nigra, VTA—ventral tegmental area, A11–13-lateral hypothalamic/incertohypothalamic catecholaminergic cell groups, A12–14-periventricular hypothalamic cell groups). The directions of the compass rose: D, dorsal; R, rostral; V, ventral; C, caudal. **(C,D)** Segmentation and volume rendering of the SN before **(C)** and after **(D)** the automated generation of an array of voxels (measuring spheres) for cell counting. **(E,F)** Segmentation and volume rendering of the A11–13 area before **(E)** and after **(F)** the automated generation of an array of voxels (measuring spheres) for cell counting. Scale bars: **(A,B)** 500 μm, **(C–F)**: 300 μm.

In the case of cell counting (Figure 1), the images containing TH+ structural elements were segmented according to voxel size and intensity, and purified from non-relevant signals (e.g., blood vessels occasionally traversing the nuclei). Then, a counting array of voxels (measuring spheres) were generated in 3D by automated scanning. Correspondence between the generated particle set and the distribution and relevance of the anatomical structures to be investigated was visually checked before each measurement, according to standards defined in preliminary experiments. Thereafter, the abundance of TH+ perikarya was determined by automated particle counting. The perikarya in the SN were defined and counted separately on each side, whereas the other regions were measured bilaterally within the common reference space straddling the midline.

**Figure 2:**
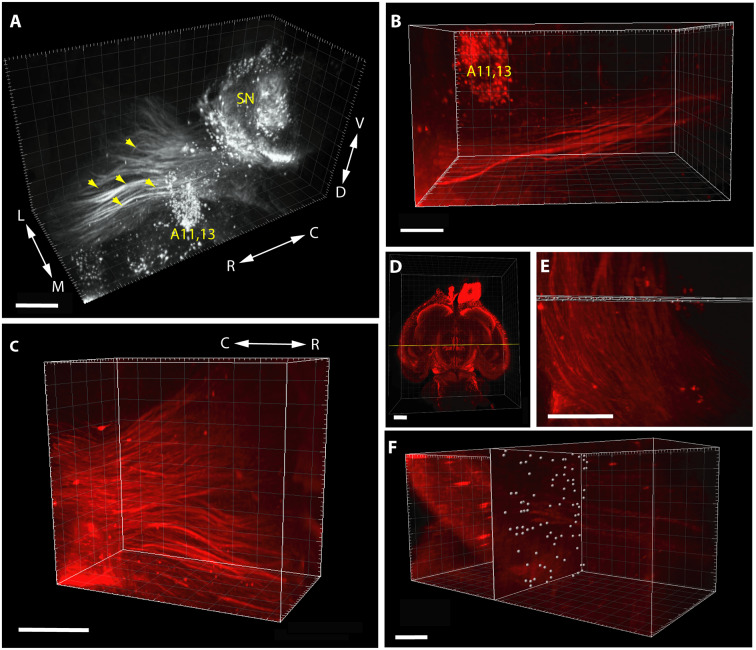
Segmentation and volume rendering of catecholaminergic diencephalic and mesencephalic cell groups together with the fascicles of mesotelencephalic dopaminergic pathway, using the iDISCO method with IMARIS, version 8.3.0, on images from representative control brain specimens immunostained for TH. **(A)** Medial view of the SN, A11–13 and the mesotelencephalic fiber tract (enhanced grayscale image). **(B)** Dorsal view of the segmented original (far-red fluorescent) image of the mesotelencephalic tract passing by the A11–13 cell group. **(C)** Oblique rostrolateral view of the mesotelencephalic pathway, note the presence of robust fascicles. **(D)** Overview image showing the position of the measuring slab (rectangular cuboid) laid at the rostral limit of A11–13, from a ventral aspect. **(E)** Enlarged view of the measuring slab following the automated generation of the array of voxels (measuring spheres) at the intersections with the fascicles of the mesotelencephalic tract. **(F)** Rostrolateral view of the measuring slab containing the automatically generated measuring spheres at the intersection windows, used for particle counting. Scale bars: **(A)** 500 μm, **(B,C,E,F)**: 200 μm, **(D)**: 1,000 μm.

It should be noted that in the case of pathway analysis (Figure 2), the whole-mount technique only permitted detection and counting of tightly apposed fiber bundles (fascicles), rather than of individual TH+ neurites (as it happens, this shortcoming could be turned into an asset, see the relevant part of “Discussion” section below). The morphometric parameter representing the abundance of measurable units of the pathway was obtained as follows. In a standard coronal plane at the rostral limit of the incertohypothalamic TH+ cell groups (A11, A13), a slab (rectangular cuboid) of a predetermined thickness (not exceeding the diameter of a single measuring sphere) was laid orthogonally to the rostrocaudal axis of the brain, to span the entire width of the MT TH+ tract. At each intersection window between this slab and the TH+ fascicles of predetermined diameter (thickness) and intensity (see above), a counting array of voxels (measuring spheres) were generated by automated scanning. One has to keep in mind that a single intersection window may generate one or more measuring spheres, depending on how massive the passing fiber bundle is. Notably, the reproducibility of the repeated scans for any given image was remarkably good at the IMARIS settings used. Following visual checking of relevance, the abundance of TH+ pathway units was determined by automated particle counting (as above). It has to be emphasized, however, that the measured “pathway units” are not meant to represent exact numbers of TH+ neurites in the tract, nevertheless, they do give a comparable and reproducible measure of the abundance of TH+ fascicles.

### Immunocytochemistry in Vibratome Sections

The brains with verified tdTomato fluorescence were embedded in 4% agarose (in PBS) and serial sections of 60–70 μm were cut in the sagittal plane. The free-floating sections were washed for 6 × 10 min in PBS containing Triton-X-100 (PBS-T), followed by blocking in 1% BSA, 0.1 M lysine and 0.1% Na-azide (in PBS-T) for 1 h at room temperature, and incubation with the primary anti-TH antibody (rabbit polyclonal IgG, Merck Millipore, catalog# AB152) diluted at 1:500 in the above blocking solution for 3 days at 4°C. After removal of the primary antibody, the sections were washed 6× (here and for all subsequent washes PBS-T was used), followed by the secondary antibody, Alexa-488 anti-rabbit IgG (raised in goat), at 1:500 in PBS-T for 1 h. After final washes (5×) the sections were counterstained with the nuclear marker fluorochrome 4,6-diamidino-2-phenylindole dihydrochloride (DAPI, Sigma-Aldrich, 5 mg/ml in PBS for 30 min at room temperature), mounted on glass slides and coverslipped under Mowiol (sealed and stabilized by nail varnish). The specimens were viewed and photographed under a Leica operating microscope with fluorescence setup, tdTomato (marking the Nkx2.1 expressing structures) appearing in its natural emission range (red), TH in green, and DAPI in blue. Selected specimens were also inspected and images acquired on a Zeiss Axio Z2 LSM 780 confocal laser-scanning microscope at 10× or 40× primary magnification. Emission spectra for each dye were limited as follows: tdTomato/570–691 nm, Alexa Fluor^®^ 488/499–570 nm, DAPI/410–499 nm. Multi-panel figures were assembled in Adobe Illustrator (Adobe Corporation).

### Immunocytochemistry in Paraffin-Embedded Sections

To determine whether VPA treatment affects the general pattern of dopaminergic and non-dopaminergic tracts, we used conventional microscopy on paraffin-embedded sections. The brains were paraffin-embedded and 10 μm sections were cut in the sagittal or coronal plane, serially mounted, and immunohistochemistry was developed as previously described (Moreno-Bravo et al., 2014). The images were collected using a LEICA MZ16FA stereomicroscope equipped with a Leica DFC550 digital camera. For immunostaining against TH, anti-TH antibody (rabbit polyclonal IgG, Merck Millipore, catalog# AB152; at 1:500) was used. For neurofilament (NF) immunolabeling, we used the antibody ABCAM AB9034 (1:800).

### Dissection of Brain Specimens for Dopamine Assay

**Figure 3:**
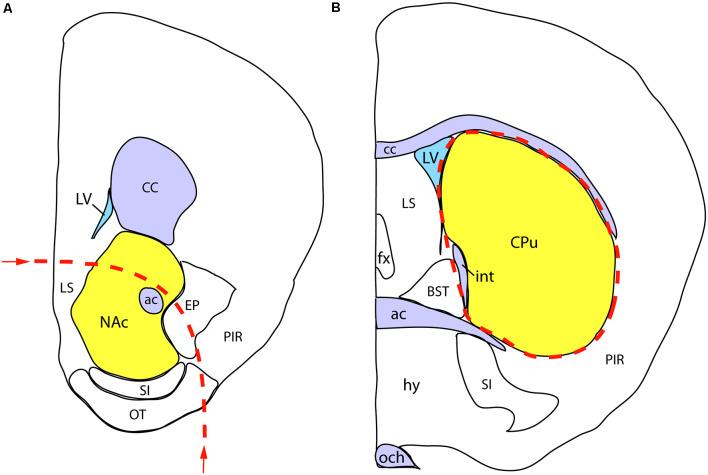
Schematic demonstration of mouse brain samples freshly dissected for dopamine assay. **(A)** The rostral face of the tissue slab 1 with the approximate borders of dissection (dashed line). The sample to be removed is centered on the nucleus accumbens (NAc), also including parts of the lateral septum (LS), endopiriform nucleus (EP), substantia innominata (SI), olfactory tubercle (OT), and the nucleus of the diagonal band (NDB; not visible at this plane of section). **(B)** The rostral face of the tissue slab 2. The dissected sample (marked by a dashed line) is centered on the caudate-putamen (CPu), also including part of the globus pallidus, external segment (not visible at this level). Other abbreviations: ac, anterior commissure, BST, bed nucleus of stria terminalis, CC, corpus callosum; fx, fornix; hy, hypothalamus; int, internal capsule; LV, lateral ventricle; och, optic chiasm; PIR, piriform cortex.

We performed the neurochemical analysis on a separate cohort of sexed P7 mice, born to mothers exposed to either VPA or saline, as described above for the whole mount experiment. Immediately after decapitation of the animals, the brains were removed, placed in ice-cold saline, and cut into coronal slabs with the help of a brain mold. Two slabs of 2 mm thickness from the appropriate coordinates were placed on a black anodized aluminum box containing ice, and the regions of interest were dissected from both sides under a stereomicroscope. The first, more rostral, the slab was centered on the NAc, but also including parts of the olfactory tubercle (TO), substantia innominata (SI), lateral septum (SL), the nucleus of the diagonal band (NDB), and the bed nucleus of stria terminalis (BST). The second, more caudal, the slab was centered on the dorsal striatum/caudate-putamen (CPu), also including part of the globus pallidus (GP). The diagram in Figure 3 shows the rostral facet of the cut slab with the approximate borders of dissection. The dissected brain specimens (the combined bilateral samples counted as one) were immediately placed into pre-weighed plastic vials, weighed, sealed, and stored at −80°C until processed further.

### Dopamine Determination by ELISA

For the quantitative measurement of tissue DA levels, we used the universal DA ELISA Kit by BioVision (#K4219-100). We followed the protocol recommended by the manufacturer, with minor modifications. Primary dilution of the tissue samples was set to 18 ml/g wet weight. (instead of 9 ml/g) to obtain enough homogenate for the duplicate measurements. This dilution was taken into account in the final calculation of results. The brain samples were homogenized in ice-cold PBS (0.01 M, pH = 7.4) by rapid sonication. Halt (protease inhibitor, Thermo Fisher, 1:100) and EDTA (Thermo Fisher, 1:100) were added to the PBS. Then we centrifuged the homogenates at 5,000× *g* for 5 min at 4°C and recovered the supernatants, from which duplicate samples (2 × 50 μl) were transferred onto the ELISA plate. Based on preliminary experiments, the standard calibration curve was expanded by interpolating two extra points, enabling more accurate reading in the expected concentration range. For the reading of the optical density, we used a Bio-Rad iMark Microplate reader at 450 nm. The protein concentration of the supernatant fraction of brain homogenates was determined using the BCA method (Olson and Markwell, 2007). Since the protein concentration values proved to be highly uniform (owing to adjustment of the primary dilutions to the wet weight of tissue), giving a mean ± SEM value of 6,992.261 ± 70.80933 μg/ml, the measured DA data were not standardized with the protein values.

### Behavioral Analysis

To ensure the long-term behavioral effects of embryonic VPA treatment in our hands were the same as for other studies, four VPA treated and four control mice were raised and tested using the standard three-chamber test procedure for social preference (Kaidanovich-Beilin et al., 2011) at P25.

### Statistical Analysis

Following prior checks on normality and homogeneity of variances, and an appropriate data transformation (log, square, cubic, or lambda) to obtain the requirements for linear models, all statistical analyses were performed using the software R. Untransformed data are visualized in the figures as mean ± SEM. The morphometric data obtained from the analysis of iDISCO specimens were compared by using a general linear model with the day of sample preparation as a random effect. The behavioral data were analysed using a general linear model. For analysis of the ELISA results, none of the data transformations fulfilled the preconditions of the linear model. To overcome this problem, we used a non-parametric repeated sampling test (R package: nparLD, Noguchi et al., 2012) on untransformed data.

## Results

### Morphology

**Figure 4:**
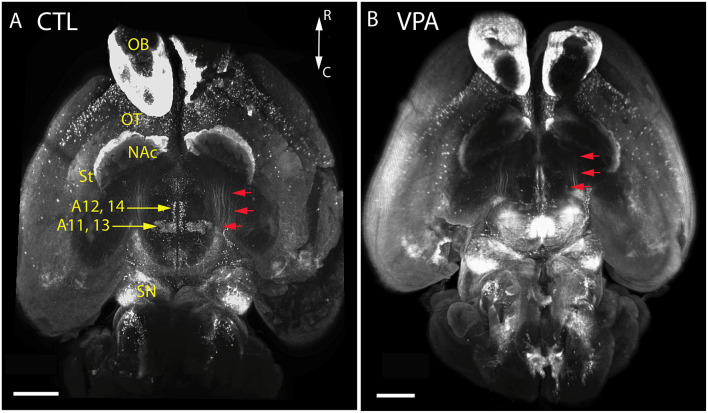
Images of whole-mount P7 mouse brains immunostained against TH, obtained using the iDISCO method of combined immunolabeling and tissue clearing. **(A)** Control (CTL) mouse born to vehicle-treated mother. **(B)** VPA-treated (VPA) mouse born to sodium valproate exposed mother. Note the apparent reduction of the volume and density in the mesotelencephalic dopaminergic pathway (red arrows) and the lateral hypothalamic/incertohypothalamic dopaminergic cell groups (A11–13) of the VPA specimen. Scale bar: 1 mm. Other abbreviations: NAc, nucleus accumbens; OB, olfactory bulb; OT, olfactory tubercle; SN, substantia nigra; St, striatum.

Qualitative comparison of whole-mount specimens of VPA and CTR P7 brains gave the impression of a reduction in the TH+ fibrous elements of the medial forebrain bundle (MFB; Figure 4, red arrows), and in the lateral hypothalamic TH+ nuclei (Figure 4, A11, A13), without obvious alterations in other TH immunoreactive regions. By employing the morphometric analysis described above in “Materials and Methods” section, this visual impression was substantiated and further specified as follows.

**Figure 5:**
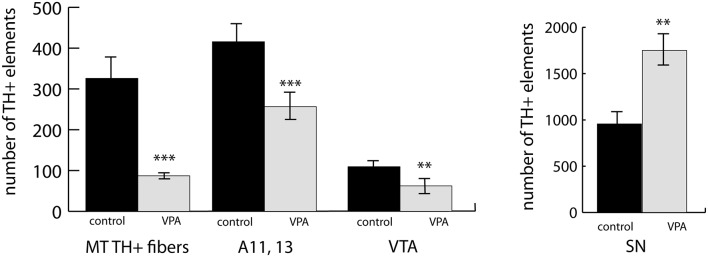
An abundance of fiber bundles (fascicles) of the mesotelencephalic dopaminergic (MT TH+) pathway and neuronal perikarya in the dopaminergic nuclei of the lateral hypothalamus (A11–13), VTA and SN, as revealed by volumetric analysis of TH-immunolabeled whole-mount (iDISCO) brain samples of control and VPA exposed P7 mouse pups. The diagrams represent the mean ± SE, *n* = 6 (control), *n* = 6 (VPA); **p < 0.01, ***p < 0.001.

We observed a marked and highly significant difference in the abundance of TH+ units of MT tract: the value of VPA exposed animals was reduced by 73.3%, i.e., to less than one-third of the value of control animals (Figure 5). Concerning the main source regions of the tract, the number of TH+ perikarya was enhanced by 83.1% in the SN but diminished by 43.0% in the VTA of the VPA exposed pups (Figure 5). Of the major hypothalamic dopaminergic centers, the lateral hypothalamic A11, together with the incertohypothalamic region, A13, showed a reduction in TH+ cell number (by 38.3%), following VPA treatment (Figure 5). Although the latter two regions, A11 and A13, were very prominent in our material, the further separation between these did not seem to be feasible in whole-mount brain tissue.

**Figure 6:**
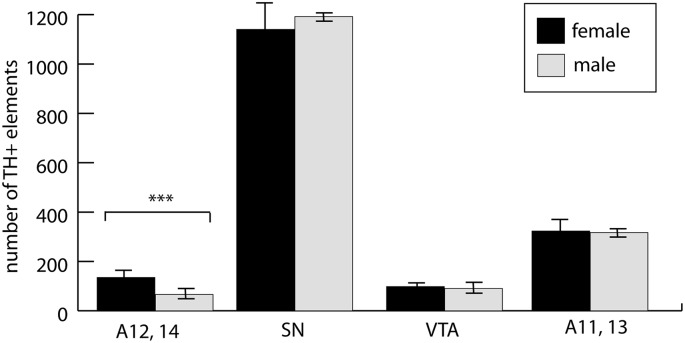
The abundance of neuronal perikarya in the dopaminergic nuclei of the lateral hypothalamus (lateral hypothalamic/incertohypothalamic A11–13 and periventricular A12–14), SN and VTA, as revealed by volumetric analysis of TH-immunolabeled whole-mount (iDISCO) brain samples of control male and female P7 mouse pups. The graph represent the mean ± SE, *n* = 7 (male), *n* = 5 (female); ***p < 0.001.

The involvement of hypothalamic dopaminergic fields raised an important question of sexual dimorphism, even though our experimental animals were still in the pre-weaning and pre-puberty phase. Although in the case of the midbrain dopaminergic nuclei, such dimorphism was only sporadically reported, and mainly observed in adults (Pappas et al., 2010), we made a systematic comparison between P7 male and female control pups. As expected, the periventricular hypothalamus, including the arcuate nucleus (collectively examined as A12, A14), was distinctly dimorphic. The number of TH+ cells in males amounted to 49.8% of the same value in females. There was no significant difference between the males and females in A11, A13, SN or VTA (Figure 6). Thus, we decided not to evaluate the effects of VPA treatment in the A12, A14 region in the mixed-sex cohort, but all the other findings (as described in the paragraph above) were confirmed to be valid effects of VPA exposure.

**Figure 7:**
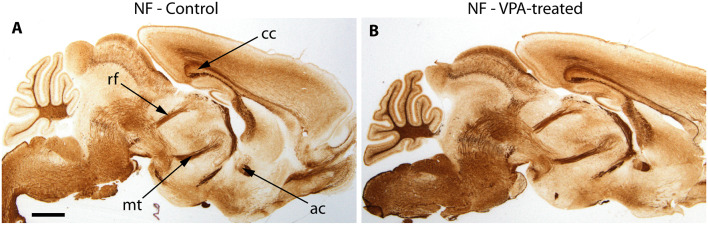
Representative matching pair of paraffin-embedded sagittal sections from control **(A)** and VPA exposed **(B)** P7 mice immunostained for neurofilament protein (NF). No difference in the morphology of major brain tracts is evident. ac, anterior commissure; cc, corpus callosum; rf, retroflex fascicle; mt, mamillothalamic fascicle. Scale bar: 1 mm.

**Figure 8:**
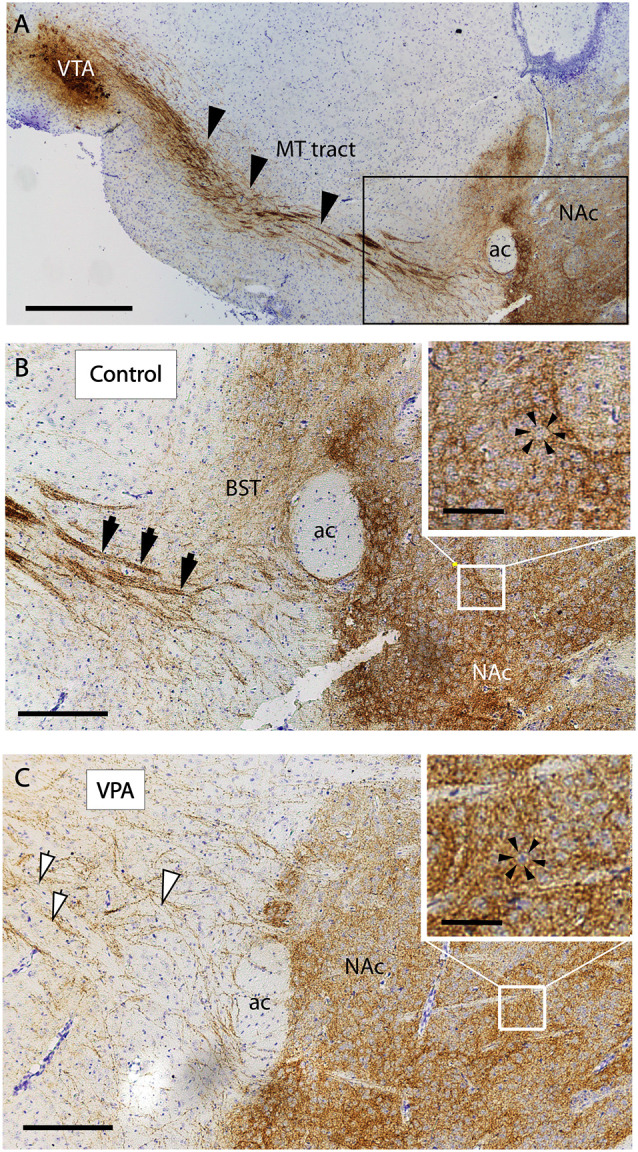
Course and distribution of the mesotelencephalic dopaminergic pathway in control and VPA exposed P7 mice. Paraffin-embedded sagittal sections immunolabeled against TH and counterstained with cresyl violet. **(A)** Low power image from control specimen, depicting the path of TH+ fibers (arrowheads) from their origin (ventral tegmental area, VTA) to the ventrobasal forebrain (i.e., centered on the mesolimbic part of the tract). Matching pair of enlarged images from control **(B)** and VPA exposed **(C)** specimens, taken at the border of the ventrobasal target area, through which the TH+ fibers pass to terminate (marked by the frame in **A**). The photomicrographs also demonstrate the difference between the two samples in the degree of fasciculation. The control displays robust fascicles (filled arrowheads), whereas the VPA is characterized by thin, less visible fascicles and more dispersed fibers (open arrowheads). On entering the target region, the TH+ fibers tend to form “dopaminergic islands,” marked by arrowheads (insets of **B,C**). Abbreviations: ac, anterior commissure; BST, bed nucleus of stria terminalis; MT, mesotelencephalic tract; NAc, nucleus accumbens. Scale bars: **(A)** 500 μm, **(B,C)** 50 μm, Inset: 20 μm.

The reported alterations of the MT pathway were not accompanied by specific morphological changes in other major brain tracts, as suggested by qualitative comparison of paraffin-embedded sections from VPA-exposed and CTR P7 brains, that had been immunolabeled against neurofilament (NF) protein. The course and dimension of the corpus callosum, fimbria hippocampi, anterior commissure, fasciculus retroflexus, and the mammillothalamic tract remained unaltered (Figure 7). As shown in Figure 8, a tendency for decreased density and reduced fasciculation of TH+ fibers could also be observed, in 10 μm sections of paraffin-embedded material, whilst the overall course of the MT pathway appeared normal in VPA-exposed animals. On entering the subpallial target region, the TH+ fibers terminate as dopaminergic islands. These displayed a similar morphological appearance in VPA and CTR brains (Figure 8).

**Figure 9:**
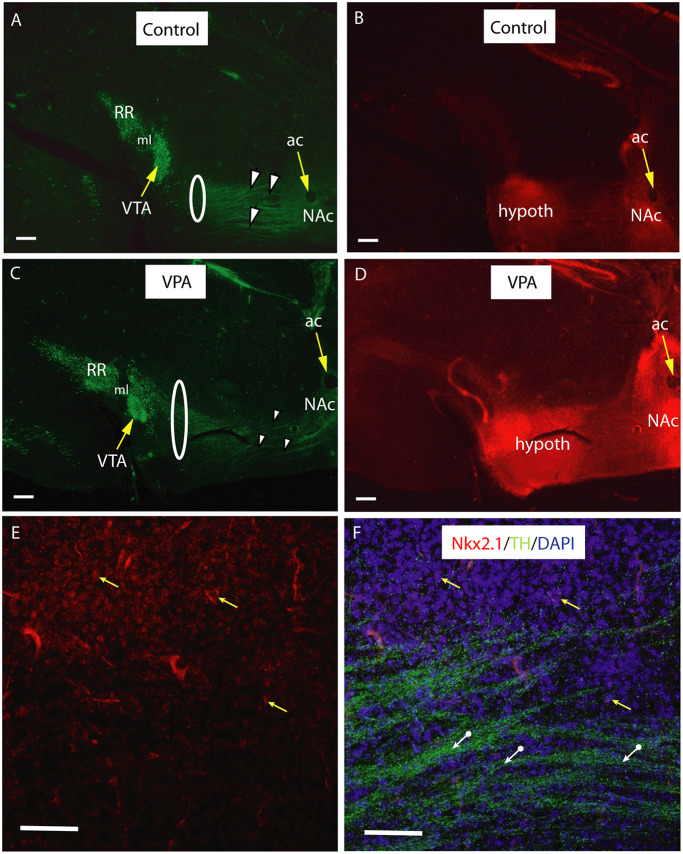
Course and distribution of the mesotelencephalic dopaminergic pathway in control and VPA exposed E18.5 mice from a tdTomato reporter line crossed with hemizygous mice expressing Cre recombinase driven by TTF-1 (Nkx2.1) promoter. Sagittal sections immunolabeled against TH (green fluorescence). Intrinsic red fluorescence of the tdTomato reporter marks the expression of Nkx2.1. For better definition, the two channels are shown as separate images. **(A–D)** Matching pairs of images from control **(A,B)** and VPA-exposed **(C,D)** mouse embryos. Note the clear separation between the VTA (A10) and the retrorubral field (A8, RR) in the source region of mesotelencephalic fibers **(A,C)**. Fasciculation of TH+ fibers traversing the Nkx2.1 + hypothalamic zone (arrowheads) is more prominent in the control specimen, as compared to the VPA-exposed specimen, whereas the same pathway appears to be wider in VPA treated samples (highlighted by the loops in **A,C**). Hypothalamic and ventrobasal forebrain localization of Nkx2.1 expression looks similar in Control and VPA specimens **(B,D)**. **(E,F)** Confocal laser scanning microscope images from a representative control section demonstrating the presence of Nkx2.1 expressing nuclei in the hypothalamic region (**E**; Channel 1 for tdTomato signal). A few examples of matching DAPI labeled nuclei (yellow arrows), and the TH+ fibers (white arrows) traversing the Nkx2.1 positive matrix are visible in (**F**; Channel 2 for the combined blue and green signals). Scale bars: 50 μm. Abbreviations: ac, anterior commissure, hypoth, hypothalamus, ml, medial lemniscus, NAc, nucleus accumbens, VTA, ventral tegmental area.

In vibratome sections (60−70 μm) of transgenic Nkx2.1^Cre/+^ − Rosa^tdTomato/tdTomato^ animals of E18.5, immunolabeled for TH, the course of the TH+ fibers passing through the Nkx2.1 expressing (tdTomato positive) matrix of the hypothalamus was particularly well demonstrated (Figure 9). Greater section thickness enabled the visualization of a wider and more dispersed path of TH+ fibers, and the decreased degree of fasciculation in VPA exposed brains, as compared to CTR specimens (Figures 9A,C). Under the confocal laser microscope, this Nkx2.1 expressing hypothalamic matrix could be resolved into individual tdTomato-positive perikarya and the traversing bundles of TH+ neurites (Figures 9E,F). The distribution of the Nkx2.1 signal, however, appeared to be similar in both phenotypes, showing prominence in two typical locations: the hypothalamus and NAc in the ventrobasal telencephalon (Figures 9B,D).

### Neurochemistry

**Figure 10:**
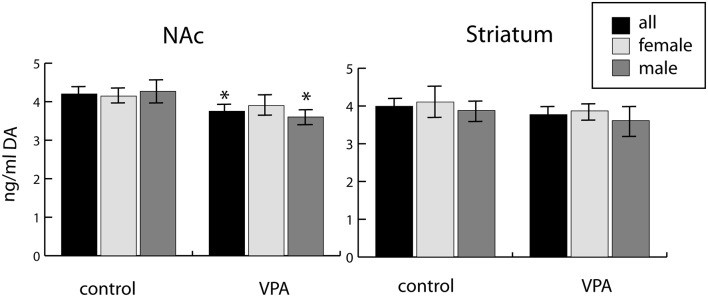
Tissue concentrations of dopamine in two selected regions (one centered on the nucleus accumbens, NAc, and the other centered on the caudate-putamen, Striatum) freshly dissected from P7 mouse brains (10 males and 18 females), as determined by ELISA assay. The data represent the mean ± SE, *n* = 18 (VPA), *n* = 10 (control), *p < 0.05.

We observed a reduction of DA in tissue samples from the ventrobasal forebrain (centered on the NAc) of VPA-exposed P7 pups, as compared to vehicle-exposed control pups. This difference was evident both in mixed-sex and all-male populations (reduction by 10.7% and 15.6%, respectively), but not significant in the all-female population (Figure 10). Conversely, no similar difference of DA content was detected in tissue samples from the dorsolateral striatum (CPu) of the same animals (Figure 10).

### Behavior

Control mice at P25 preferred an unfamiliar conspecific male over an empty compartment in the standard three-chamber test, while VPA treated mice spent more time in the empty chamber (Supplementary Figure S1).

## Discussion

### Summary and Interpretation of Current Findings

We demonstrated a marked reduction of the number of detectable units constituting the mesotelencephalic dopaminergic (TH+) pathway in 7-day-old mouse pups born to VPA-exposed mothers, as compared to those born to vehicle-exposed mothers. This finding could either be ascribed to a genuine deficiency of neurites, reduction of TH immunoreactivity, or a redistribution of fibers. As for the first option, we did not observe changes in the size and conformation of the major brain pathways, including the MFB, in paraffin-embedded sections labeled with NF, and only sporadic reports support such an overall reorganization of brain morphology following VPA exposure. Kuwagata et al. (2009) described an abnormal pathway in the pons (though the dose and timing of VPA administration were distinctly different from those used in the present study). A size reduction of the anterior commissure has been reported in the marmoset following VPA exposure, based on diffusion tensor imaging (Mimura et al., 2019). Another indirect argument against the occurrence of a massive overall loss of neurites could be our observation that the tissue level of DA in the dorsolateral striatum (CPu) remained unchanged (present study). Given the considerable contribution of the mesostriatal pathway within the MFB (Nieuwenhuys et al., 1982), this could hardly be the case if ca. two-third of all DA neurites was truly missing from the tract. However, a genuine reduction of DA did take place in the ventrobasal forebrain, where a partial loss of TH+ fibers in the mesolimbic component of the pathway was observed. Ventral mesencephalic input to the ventrobasal forebrain nuclei (also included in our tissue specimens assayed for DA) comes predominantly from the A10, as evidenced by the following report. In a detailed tracing study on rats, Hasue and Shammah-Lagnado (2002) found the percentage of A10 afferents to be 80–90% in the accumbens shell, and nearly 50% in the BST (a further 10–30 percent of A10 afferents being shared with dorsal raphe (DR) or the periaqueductal gray (PAG), but, in each case, the contribution of the A9 (SN) to the dopaminergic innervation of these regions was negligible (Hasue and Shammah-Lagnado, 2002; for a detailed pattern of the mesoaccumbens pathway in the mouse see Rodríguez-López et al., 2017). A more precise description of the missing neurites is impractical because the fibers of VTA origin are not confined to a separate and sufficiently narrow zone of the MFB (Veening et al., 1982).

Considering the possibility of reduced TH immunoreactivity in the existing fibers, previous reports have shown that TH immunolabel precedes (by 1–2 days, in the rat) the appearance of catecholamine fluorescence or the immunolabel for DA in the cells and fibers (Specht et al., 1981; Voorn et al., 1988). Thus, once the neurites are present, they should express TH. This makes a selective loss of immunoreactivity highly unlikely, in our case.

In so far as the third explanation is concerned, previous studies have already indicated a reorganization of the MT pathway in Wnt5a KO mutant mice (Blakely et al., 2011), with disrupted fasciculation of DA axons *en route* to their targets. In the cited study, the TH+ fibers in the MFB appeared more loosely arranged in KO mutants than in WT mice, with a denser arborization of terminals in the striatum. Other decisive factors in the fasciculation and channeling of the mesostriatal dopaminergic pathway are Semaphorine 3F (Sema3F) and its receptor Neuropilin-2 (Nrp2), mainly as early chemorepellents (Hernández-Montiel et al., 2008; Kolk et al., 2009; Yamauchi et al., 2009). The authors observed defasciculation and widening of the MFB in Nrp2 or Sema3F KO mice, together with an aberrant growth of fibers (Hernández-Montiel et al., 2008; Kolk et al., 2009; for a comprehensive review of the issue see Brignani and Pasterkamp, 2017). Severe pathfinding errors in mesodiencephalic dopaminergic axons have been noted also in Robo1/2 and Slit 1/2 KO mice, resulting in a wider-than-normal path of fibers (Dugan et al., 2010). However, such misprojection of DA fibers may be due, at least in part, to the aberrant growth of descending GAD65-positive striatonigral neurites that precede and act as a scaffold for the rostrally directed growth of DA fibers (García-Peña et al., 2014).

Similarly, in the present study, the anatomical finding in VPA exposed brains was consistent with dispersion and partial defasciculation of TH+ fibers, leading to an apparent widening of the TH+ tract by E18.5 (Figure 9). Presumably, it is precisely this perturbed fasciculation and the aberrant course of MT TH+ fibers that made the detection of such prominent changes possible when using the whole-mount technique. A fortuitous combination of the technical advantage of the iDISCO method (3D imaging in full transparency) and its apparent “drawback” (only the fascicles, but not single neurites, can be visualized and counted as individual units), together with the appropriate time window of detection (P7), enabled us to hit upon a meaningful disturbance in the development of DA pathways.

For the counting of the DA cells, the advantage of the measuring system could be exploited without limiting considerations. Remarkably, in the VTA the number of TH+ cells was diminished in VPA treated brains (in apparent agreement with the decrease of tissue DA in the ventrobasal forebrain), but in the SN the effect was the opposite: the number of TH+ cells was increased (with an unchanged level of tissue DA in the main target area, CPu). This raises the possibility that part of the effect of VPA could be abnormal segregation and/or displacement of the DA precursor cells from their final position in the midbrain. Thus, the VPA challenge at E13.5 might have specifically affected both the formation and placement of VTA neurons, since these (at least in the ventromedial VTA) are born and differentiated later than those of the SN (Blaess et al., 2011). However, the increased abundance of TH+ perikarya in SN following VPA exposure (as observed in the present study) may also reflect a compensatory hyperproliferation associated with the aberrant growth of the neurites emanating from these cells. In any case, our finding of an increased number of neurons in the SN argues strongly against the possibility of neurodegeneration underlying the observed effects of VPA. Neuroproliferative and neuroprotective effects of VPA, for the DA system, have been reported previously, e.g., promoting TH expression in cultured neural progenitor cells and brain transplants (Yoshikawa et al., 2013) or preventing the loss of dopaminergic neurons of SN in neurodegenerative models of parkinsonism (Kidd and Schneider, 2011; Carriere et al., 2014).

The diencephalic TH+ cell groups A12, A14 proved to be sexually dimorphic in the age groups we investigated, in agreement with previous reports (Arbogast and Voogt, 1991; Balan et al., 2000) and were, therefore, not considered for VPA dependent effects. However, no sexual dimorphism was evident in the TH+ cells of the lateral hypothalamic/incertohypothalamic region (A11, A13) at P7 (but see Pappas et al., 2010, which reports distinct sexual dimorphism in the A11 of adult mice), and their number was also reduced after VPA treatment. This would indicate that the development of this cell group may not have been completed by E13.5, i.e., the time of VPA administration. Notably, the TH+ cells of the latter region are of mixed catecholaminergic type, e.g., about 60% of the TH+ neurons coexpress calcitonin in A13 (Leclercq and Herbison, 1996), meaning that the effect of VPA may not be confined to the purely dopaminergic phenotype of ventral mesencephalic (VM) neurons.

Development and reorganization of dopaminergic innervation of the striatal and accumbal target areas, including final arborization and pruning of synapses, is not yet finished at P7 (Voorn et al., 1988; Lesting et al., 2005). The patchy appearance of the border region of the ventrobasal forebrain invaded by the TH+ neurites (representing dopaminergic islands) was still evident at P7 (Figure 8). This anatomical phenotype remained unaltered in VPA-exposed mice. Nor did we find an alteration in the pattern of Nkx2.1 expression (in either typical location: basal telencephalic or hypothalamic) in E18.5 embryos of VPA exposed Nkx2.1^Cre^ mothers. The latter transcription factor (presumably *via* Slit mechanism) is known to participate in the axon guidance of the ascending (mesotelencephalic) tract, as evidenced by the aberrant path and decussation of TH+ fibers in Nkx2.1^−/−^ mutants (Marín et al., 2002).

### The Relevance of Findings for the Development of Dopaminergic Pathways in Rodents, and the Critical Time Window of VPA Administration

Reports on anatomical changes in the dopaminergic system following VPA are remarkably sparse. VPA administration at E12.75 caused perturbed compartmentalization (i.e., decreased μ-opioid receptor-positive striosomes, but increased calbindin-positive matrix) in the rostral striatum of mice (Kuo and Liu, 2017). Aberrant migration of TH+ and 5-HT+ neurons, and the appearance of an abnormally running nerve tract in the pons were observed after an early (at E11) gestational exposure of rats to a rather high dose (800 mg/kg) of VPA (Kuwagata et al., 2009).

Following the proliferation, early migration, and distribution of VM dopaminergic neurons, the cells extend neurites bound for their targets (Str, NAc, prefrontal cortex) to form the mesotelencephalic tract. Within the MFB the dopaminergic axons group into bundles (fasciculation), preventing them from getting diverted while passing through the hypothalamus. The process requires carefully timed regulation. Several signaling molecules are known to play a role in the guidance of dopaminergic pathways. One of the factors implicated in the development of the mesotelencephalic pathway is the Wingless-type signal molecule Wnt5a, which specifically affects dopaminergic neuritogenesis (mediated by planar cell polarity receptors Frizzled; Fenstermaker et al., 2010). This protein is a repellent factor for DA neurites, and in Wnt5a deficient mutants, disrupted fasciculation of the mesotelencephalic TH+ fibers is accompanied by an increase of pathway volume (Blakely et al., 2011). A similar regulatory function has been ascribed to Wnt7a, which is mediated by the β-catenin/canonical pathways (Fernando et al., 2014). Netrin-1 and its receptor “deleted from colorectal cancer” (DCC) have also been confirmed as potential guidance factors (Volenec et al., 1998; Osborne et al., 2005; Li et al., 2014). Additionally, it has been suggested that Semaphorin 3A and 3F participate in navigating DA axons to their target areas (Torre et al., 2010). The role of the cell adhesion molecule CHL1 in early DA progenitor migration and differentiation has also been demonstrated (Alsanie et al., 2017). Notably, *Chl1* has been described among the candidate risk genes in ASD families of humans (Salyakina et al., 2011). Further studies have described the contribution of the Slit—Robo system in pathfinding by mesodiencephalic dopaminergic axons (Marillat et al., 2002; Lin et al., 2005). Recently, a novel mechanism of molecular interaction between the cell adhesion molecule ALCAM (CD166) and the Semaphorine receptor complex has been reported to selectively orchestrate the growth of midbrain DA axons (Bye et al., 2019).

Concerning early differentiation and segregation of midbrain dopaminergic neurons, Shh and Gli1 are the most prominent factors to determine which cells should constitute the future SN or VTA. This conclusion is based on the rostrocaudal topology and early/late expression of the above factors (Blaess et al., 2011; Hayes et al., 2011). Based upon ^3^H-thymidine incorporation and fate mapping studies, the precursors of ventral mesencephalic (VM) DA neurons get separated first from those of the red nucleus between E8.5–E10.5, and the DA neurons are born, in the mouse, between E10.5 and E14.5. The specific generation of the precursors of SN and VTA is completed by E12.5 (Bayer et al., 1995; Blaess et al., 2011). In mice, the first DA neurites appear on E11.5. Initially, they project dorsally, then they turn in a rostral direction, heading for their forebrain targets by crossing the hypothalamus (Blakely et al., 2011). The first DA neurites reach the border of ganglionic eminence at E14.5 and a distinct DA tract in the MFB is evident by E16. Then, the fibers enter the lateral ganglionic eminence (LGE), first targeting the Str (E16–17) then the NAc (E16–17), followed by the prefrontal cortex (E18) and the neocortex (E19 onwards). Dopaminergic innervation of these regions will have been completed by birth. However, the formation of synaptic contacts throughout the LGE and cortex, with axonal branching, pruning, and synaptogenesis, continues into the first weeks of postnatal development, in which we have observed changes. Thus, the administration of VPA on E13.5 (as in the present study) fell on a critical point of development: well after the formation of dopaminergic cell groups of VM and the onset of axonal growth, but before the formation and consolidation of the mesotelencephalic pathway.

### Putative Molecular Factors Underlying Prenatal Exposure to VPA

The precise mechanisms by which the VPA model leads to behavioral changes are not well understood. VPA is known to affect transcription, cation channels, and excitatory/inhibitory balance, as described below.

#### Transcriptional Effects

The mechanism of action of VPA is a rather complex one, but it is likely that it mainly elicits developmental and behavioral alterations by inhibition of histone deacetylase (Moldrich et al., 2013), potentially affecting gene expression and transcription. It engages, among others, the Wnt1 signaling pathway, a robust “caudalizing” factor in the rostrocaudal patterning of CNS (Wiltse, 2005; Jang and Jeong, 2018). Prenatal VPA treatment altered the expression in neural stem/progenitor cells of genes associated with cell migration, including CXC motif chemokine receptor 4 (Cxcr4), leading to the ectopic localization of newborn neurons in the hilus of the hippocampus (Sakai et al., 2018). Importantly, of the 20 genes found to be downregulated in VPA-exposed marmoset neonates, Frizzled3 (FZD3) and PIK3CA have been implicated in the axon guidance signaling pathway required for the normal development of the anterior commissure and *corpus callosum* (Mimura et al., 2019).

#### Effects on Excitatory/Inhibitory Mechanisms and Cation Channels

Concerning the role of the GABA system, prenatal VPA was found to reduce GABAergic synaptic inhibition in the temporal cortex of rats (Banerjee et al., 2013). Following this, an increase of excitation/inhibition ratio was reported in the afferents to serotonergic neurons of the dorsal raphe nucleus in male rats prenatally exposed to VPA. Elevation of the spontaneous firing of 5-HT neurons may account for the enhanced anxiety and stereotypies observed in prenatal VPA-exposed rats (Wang et al., 2018). The known excitatory/inhibitory imbalance in the autistic brain has been attributed to altered expression of transcription factors governing glutamatergic/GABAergic differentiation during fetal development (Kim et al., 2014). The latter authors found a transient elevation of Pax6 expression following treatment with VPA or other histone deacetylases (HDAC) inhibitors, due to an increased acetylated histone binding in the Pax6 promoter region, which resulted in enhanced expression of glutamatergic proteins in the offspring postnatally (Kim et al., 2014).

The selective vulnerability of midbrain DA neurons in PD may be determined by the hyperpolarization-activated cyclic nucleotide-gated (HCN) cation channels and their encoding genes (He et al., 2014; Chang et al., 2019). VPA was found to increase the expression of HCN-1 in the forebrain of mice (Lauber et al., 2016). Sodium channels can also be modified or downregulated by VPA (Tan et al., 2017; Zanatta et al., 2019). The observed attenuation of cation channels seems to be more relevant for the anticonvulsive action of VPA, than for the dopaminergic deficits reported by us.

### Clinical Translation of Findings. Role of DA in Neuronal Development and Axon Guidance Mechanisms

Several molecular mechanisms, potentially relevant for the differential vulnerability of midbrain DA neurons, are under investigation, mainly about PD (see the critical review by Brichta and Greengard, 2014). Our study describes the effects of a prenatal, developmental intervention, not a pathological process leading to PD in the adult brain. Moreover, any differences found between the DA cells of SN and VTA have always supported the survival of VTA and degeneration of SN (see Brichta and Greengard, 2014). In our case, the very finding seems to be the opposite: reduced VTA and enhanced SN.

The assumption that dopaminergic innervation might play an organizational role in striatal development was formulated about 30 years ago, necessitating a detailed description of the ontogeny of relevant dopaminergic centers and pathways (Voorn et al., 1988). On E17 a prominent bundling of DA neurites is observed in rats (Voorn et al., 1988). During the late prenatal and early postnatal days, the DA fibers first appear as “patches” (also termed dopaminergic islands) in the target zones of the striatum, which get dissipated in the following weeks of postembryonic development (by the end of week 3). Lack of persistence and failure of the continued growth of such islands were found to be critical alterations in the weaver mutant (Roffler-Tarlov and Graybiel, 1987). The relatively late appearance of dopamine immunoreactive, symmetrical axospinous synapses in striatal and accumbal targets (Antonopoulos et al., 2002) indicates that the development of the dopaminergic system may contribute to the organization (pattern formation) of the striatum and NAc. Such a late postembryonic development of striatum is not exclusive to rodents. The striatum of the rhesus monkey takes more than 12 months for an adult-like pattern of neuropeptide expression to be attained, and this dynamic process also requires synchronous and compartment-selective changes in afferent innervation (Martin and Cork, 2014). By analogy, in recent PET studies of human PD, altered wiring (derangement of nigrostriatal—to a lesser degree, of ventral tegmentostriatal—axons) was found to be the initial step in neurodegeneration (Caminiti et al., 2017). Genetic disturbances related to the dopaminergic system, underlying certain forms of human ASD, have been reported previously, e.g., *de novo* missense mutation of the dopamine transporter gene (Hamilton et al., 2013), or the involvement of dopamine-3-receptor gene (DRD3; Staal et al., 2012). For an overview see the *in silico* analysis by Nguyen et al. (2014).

Clearly, the dopaminergic pathway is not the only system to undergo cell proliferation, migration, and neurite growth at E13–14, the time window of VPA administration. Nevertheless, certain defects of the dopaminergic system are known to be associated with disorders of decision making and sociability, both in experimental animals and in humans. Based on the occurrence of such symptoms in ASD, it has been suggested that an impaired dopaminergic system might be a causal factor in certain forms of the disease (Bissonette and Roesch, 2016; Alsanie et al., 2017). The coherent neuroanatomical and neurochemical results of the present study point in the same direction: diminished dopaminergic input to the ventrobasal telencephalon from a critical time point of embryonic development (E13-E14) onwards may hinder the maturation and patterning of certain brain centers earmarked for decision making and sociability, and cause the observed (Supplementary Figure S1) impairment in social preference. If modified to a variable degree by compensatory mechanisms, some of the early defects may prove to be irreversible and, in the long run, may lead to lasting changes of social adaptation, potentially modeling those evoked *in utero* by similar, sharply timed pathogenic events in the etiology of human ASD.

## Data Availability Statement

The datasets generated for this study are available on request to the corresponding author.

## Ethics Statement

The animal study was reviewed and approved by Food Chain Safety and Animal Health Directorate of the Government Office for Pest County, Hungary (license number: XIV-I-001-2269-4/2012) and Universidad Miguel Hernãndez OEP Animal Experimentation Committee (code 2014/VSC/PEA/00166).

## Author Contributions

ÁÁ carried out the experiments on whole mount preparations, by doing part of the laboratory work and the image analysis and evaluation of iDISCO specimens. RK did the ELISA assays for dopamine. VC, RM-R and IJ carried out the immunohistochemistry studies on wild type and Cre-recombinant mice. GZ and LG participated in the ELISA assays and in the statistical evaluation of datasets. DE, EP and SM contributed to experiment planning and organization, to the microscopic evaluation of histological specimens, and to the final form of manuscript. AC did most of the experiment planning, with an active participation in the preparation and evaluation of microscopic and ELISA samples, and wrote the article.

## Conflict of Interest

The authors declare that the research was conducted in the absence of any commercial or financial relationships that could be construed as a potential conflict of interest.

## Footnotes

^1^http://bitplane.com
