# Supplementary material for: Gestational Exposure to Sodium Valproate Disrupts Fasciculation of the Mesotelencephalic Dopaminergic Tract, With a Selective Reduction of Dopaminergic Output From the Ventral Tegmental Area
Source: Front Neuroanat. 2020 Jun 5;14:29. doi: 10.3389/fnana.2020.00029 (PMC7290005; doi:10.3389/fnana.2020.00029)
Supplement: Supplementary file 1 [file Data_Sheet_1.PDF]

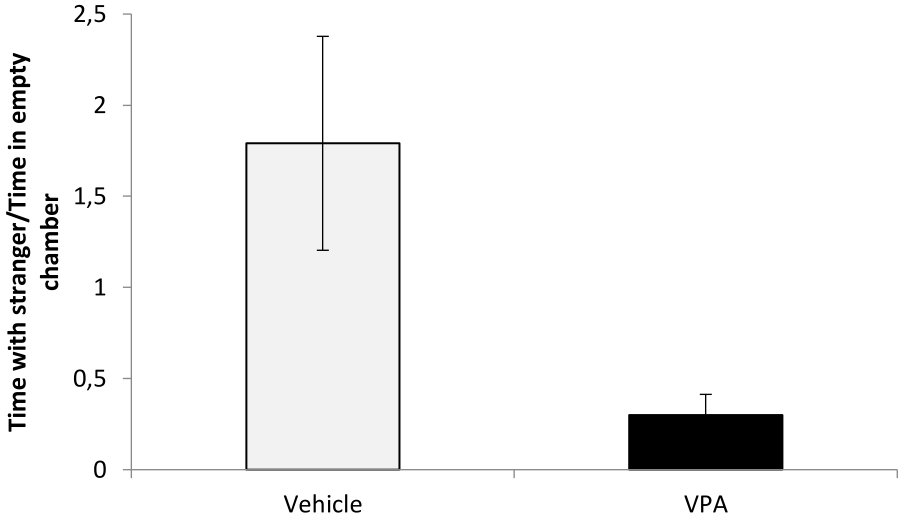

Figure S1. VPA treated mice showed no preference toward a conspecific compared to the controls ($F_{1,3}=6.22$, $p=0.047$) in the three-chamber test (Kaidanovich-Beilin et al. 2011).
